# Rationale for Prostate-Specific-Membrane-Antigen-Targeted Radionuclide Theranostic Applied to Metastatic Clear Cell Renal Carcinoma

**DOI:** 10.3390/ph16070995

**Published:** 2023-07-12

**Authors:** Anne Laure Giraudet, Armelle Vinceneux, Valentin Pretet, Emilie Paquet, Alicia Sanchez Lajusticia, Fouzi Khayi, Jean Noël Badel, Helen Boyle, Aude Flechon, David Kryza

**Affiliations:** 1 Centre Léon Bérard, 69008 Lyon, France; armelle.vinceneux@lyon.unicancer.fr (A.V.); valentin.pretet@lyon.unicancer.fr (V.P.); emilie.paquet@lyon.unicancer.fr (E.P.); alicia.sanchez-lajusticia@lyon.unicancer.fr (A.S.L.); fouzi.khayi@lyon.unicancer.fr (F.K.); jean-noel.badel@lyon.unicancer.fr (J.N.B.); helen.boyle@lyon.unicancer.fr (H.B.); aude.flechon@lyon.unicancer.fr (A.F.); 2 Lumen Nuclear Medicine Department, Hospices Civils de Lyon, 69437 Lyon, France; 3 UNIV Lyon—Université Claude Bernard Lyon 1, LAGEPP UMR 5007 CNRS Villeurbanne, 69100 Villeurbanne, France

**Keywords:** radionuclide therapy, metastatic clear cell renal carcinoma, PSMA

## Abstract

Prostate-specific membrane antigen (PSMA), whose high expression has been demonstrated in metastatic aggressive prostate adenocarcinoma, is also highly expressed in the neovessels of various solid tumors, including clear cell renal cell carcinoma (ccRCC). In the VISION phase III clinical trial, PSMA-targeted radioligand therapy (PRLT) with lutetium 177 demonstrated a 4-month overall survival OS benefit compared to the best standard of care in heavily pretreated metastatic prostate cancer. Despite the improvement in the management of metastatic clear cell renal cell carcinoma (mccRCC) with antiangiogenic tyrosine kinase inhibitor (TKI) and immunotherapy, there is still a need for new treatments for patients who progress despite these drugs. In this study, we discuss the rationale of PRLT applied to the treavtment of mccRCC.

## 1. Introduction

The estimated number of new cases of kidney cancer in the world in 2020 was around 431,288 new cases, as reported in GLOBOCAN. Kidney cancer thus represents about 2% of all cancers [1]. It affects twice as many men as women. The average age at diagnosis is 66 years old. Kidney cancer is associated with several risk factors, the main ones being smoking, overweight or obesity, hypertension, and dialysis treatment for over three years.

Kidney cancer is associated with a good prognosis when diagnosed at a localized stage, which is the case in more than half of patients. For this group, the 5-year survival rate is 93%. The prognosis is poorer when the cancer is diagnosed at a later stage. If kidney cancer has spread to the surrounding tissues or organs and/or the regional lymph nodes, the 5-year survival rate is 70%. If the cancer has spread to a distant part of the body, the 5-year survival rate is 13%. In 2020, GLOBOCAN found that kidney cancer was responsible for 179,368 deaths around the world.

Clear cell renal cell carcinoma (ccRCC) represents 60–75% of renal cell carcinomas [2]. Most cases are sporadic (95% of all cases). The remainder of cases are related to hereditary von Hippel–Lindau disease and various other familial diseases. With papillary and chromophobe subtypes, they represent more than 90% of all RCC. ccRCC is one of the most aggressive subtypes of kidney cancer. Up to 30% of ccRCC patients have metastases at the time of diagnosis, and ~60% have metastases within 2 to 3 years of initial diagnosis. Metastases are the major cause of mortality associated with ccRCC.

The treatment of metastatic ccRCC is based on targeted agents such as antiangiogenic tyrosine kinase inhibitors (VEGF receptor tyrosine kinase inhibitors, VEGFR TKI) as metastases are highly vascularized due to the overproduction of platelet-derived and vascular endothelial growth factors (PDGFs and VEGFs). There is also a long rationale for using immunotherapy in ccRCC, and more recent checkpoint inhibitors (ICIs) have been studied in ccRCC. They have shown interesting clinical activity in second-line treatment as monotherapy and in first-line treatment in combinations (ICI+ICI or ICI and antiangiogenic TKI). In advanced ccRCC, treatment options are based on the International Metastatic RCC Database Consortium (IMDC) prognostic groups and supported by large phase III trial results. TKI monotherapy can be used in some favorable risk patients. Combinations of a VEGF-targeted agent plus an immune check point inhibitor can be used for patients from any prognostic group and a combination of nivolumab plus ipilimumab for intermediate and poor prognosis groups.

Ipilimumab in combination with nivolumab can be used in patients with intermediate/poor risk disease. Median progression free survival (PFS) in the CHECKMATE 214 was 11.2 months (IC95% 8.4–16.1), and median overall survival was (OS) 48.1 months (IC95% 35.6-NE) in the intermediate/poor risk group [3].

The combination of pembrolizumab plus axitinib was the second combination regimen to be authorized. The KEYNOTE426 study [4] randomized patients between sunitinib and the combination of axitinib 5 mg × 2 and pembrolizumab every 3 weeks. The update with a median follow-up of 42 months found a significant increase in OS. The median PFS was 15.4 months and the overall response rate was 60% in the combo arm.

The combination of cabozantinib and nivolumab in the front-line setting was supported by data from the CheckMate 9ER study that compared it to sunitinib [5]. The results showed a significant OS advantage for cabozantinib and nivolumab [(hazard ratio (HR) 0.56; *p* < 0.001)]. Reponses rates and PFS also significantly favored the combination (56% versus 27% and HR 0.51, 95% CI 0.41–0.64, respectively).

More recently, data from the CLEAR study were published for pembrolizumab associated with Lenvatinib, with an impressive response rate of 71%, with 16% complete response and a median PFS of 23.9 months, becoming a new option for first-line treatment [6].

Currently, most patients are treated with a combination as first-line treatment. Some new drugs such as the HIF-2α inhibitor belzutifan are entering the landscape of ccRCC treatment with already some results in monotherapy or in combination with TKI [7]. Imaging patients with [18F]FMISO PET or [18F]FAZA PET could be helpful to select patients for belzutifan as their uptake correlates with hypoxic cells [8]. The strategy for the use of HIF inhibitors still needs to be defined.

Currently, for patients who did not receive a TKI in first-line treatment, because they received the nivolumab–ipilimumab combination, second-line treatment is a TKI. For other patients, who received an ICI/TKI combination, the issue will be to find which sequence will be optimal. The ESMO recommendations propose a TKI which has not been given previously [9]. In this setting, the response rates are around 20%, and the level of IIIB recommendation is identical for all the molecules mentioned.

Some ongoing studies aim to assess the benefit of continuing immunotherapy during second-line treatment. The continuation of immunotherapy is therefore not recommended outside of a clinical trial. In addition to the therapeutic impact, it will also be necessary to look at the risk–benefit balance of added toxicities and financial impact, which cannot be neglected. Inclusion in a clinical trial should be considered at this stage, either to help develop the sequence strategy or to test new molecules.

The data on second- and third-line therapy for patients with metastatic ccRCC who have been treated with combinations remain limited. It is therefore important to develop new therapies to offer to patients with metastatic ccRCC.

## 2. PSMA

**Figure 1:**
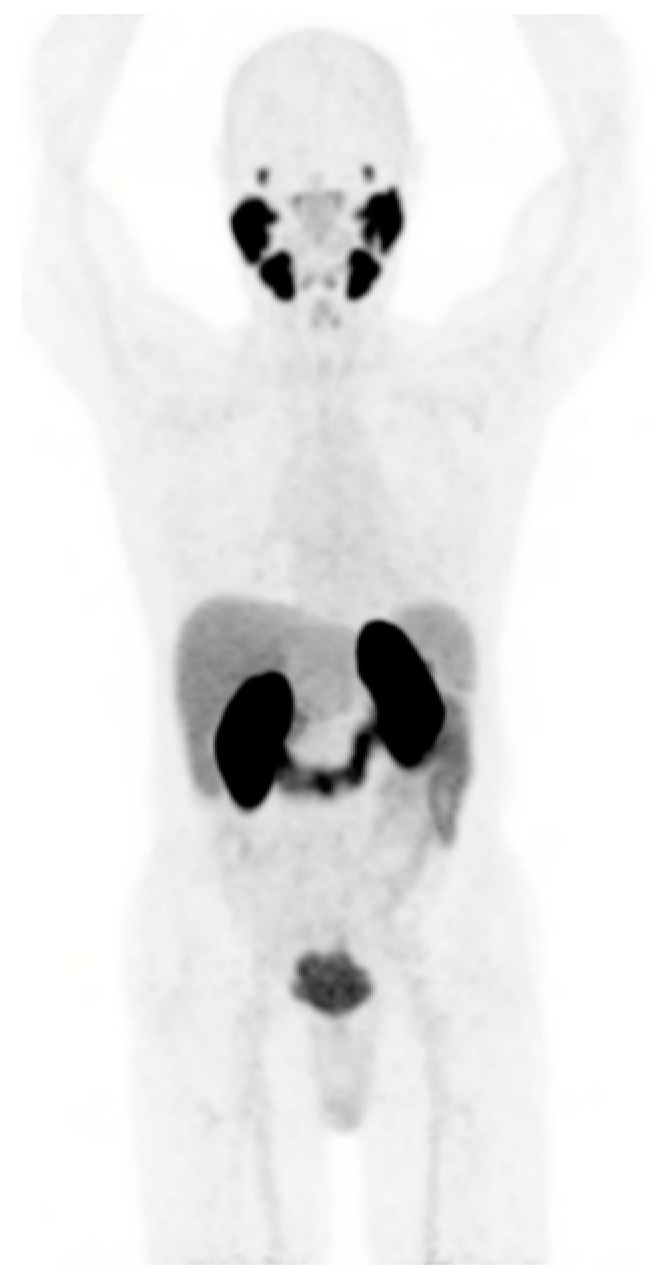
Physiological PSMA expression in normal tissues assessed by [68Ga]Ga-PSMA-11 PET performed at Lumen nuclear medicine department.

Prostate-specific membrane antigen (PSMA) is highly expressed on the membrane of most prostate cancer cells in correlation with Gleason score and castrate resistance status, and to a lesser extent in normal tissues (prostate, small intestine, salivary and lachrymal glands, and kidney) [10]. This explains the human physiological biodistribution of radiolabeled PSMA ligands used for PET imaging (PSMA PET) (Figure 1).

PSMA is a non-secreted membrane enzyme. It is a carboxypeptidase and folate hydrolase, with a large extracellular domain. It plays an oncogenic signaling role in prostate cancer cells, acting on glutamate receptors and activating the Pi3K and Akt pathways [11]. It also promotes endothelial invasion by hydrolyzing small peptides of the extracellular matrix. One of these hydrolysis products, the dipeptide leu-gln (LQ), promotes angiogenesis by activating the integrins α2β1 and α3β1 [12]. High PSMA expression has been demonstrated in neovessels of various solid tumors, reflecting neoangiogenesis, including renal cell carcinoma (RCC) [13,14].

## 3. PSMA in ccRCC

PSMA expression in RCC endothelial cells depends on the histopathology subtype. It is mainly overexpressed in clear cell RCC (ccRCC), with 86–100% of tumors being positive in immunohistochemistry compared to 30–61% of chromophobe RCC and 0–28% of papillary RCC [15]. ccRCC demonstrates a diffuse staining pattern which is in most cases consistent with the highly vascularized characteristics of ccRCC [16]. Indeed, only ccRCC relies on the VHL mutation responsible for HIFs stabilization and accumulation, leading to neoangiogenesis through the stimulation of hypoxia response elements [17].

In the treatment of 257 naïve patients presenting with renal carcinoma, immunohistochemistry on primary kidney lesions demonstrated an intense PSMA expression greater than normal renal parenchyma [18]. Of the 30 patients with metastatic ccRCCc, the primary tumor was PSMA-positive in 29 cases (96.7%). Of a higher grade and stage, metastatic and lethal ccRCC showed higher PSMA expression in tumor vessels, reflecting tumor prognosis. On univariate and multivariate analysis, the intensity of positive vs. negative endothelial PSMA protein expression was significantly associated with overall survival, with a median follow-up exceeding 10 years.

## 4. PSMA as a Target for Theranostics: From Imaging to Therapy

Theranostics is an emerging field in which diagnosis and specific targeted therapy are combined to achieve a personalized treatment approach for the patient. As PSMA expression is linked to tumor neo-vascularization in many different types of tumors, PSMA PET may be used to select patients and predict response to antineoangiogenetic therapy [19]. In ccRCC, PSMA expression appeared to correlate with VEGFR-2 and PFGFR-β overexpression [20], and a repeated PSMA PET after 1 month of Sunitinib treatment was superior to conventional CT in detecting early treatment response in one mccRCC patient [21]. PSMA PET could also detect early treatment response after cabozantinib [22].

PSMA PET may help to select patients for immunotherapy. Indeed, the polybromo-1 mutation (PBRM1) observed in 40% of ccRCC induces neoangiogenesis. It is correlated to tumor stage and aggressivity as well as cytotoxic T-lymphocytes infiltration of the tumor microenvironment. As PBRM1 tumors tend to better respond to PD-1 targeting immunotherapy, PSMA PET may be predictive of treatment response [23].

In nuclear medicine clinical practice, theranostics consists of a two-step procedure from diagnosis to radionuclide therapy. We use the same ligand (specific of a tumor target), labeled with a Gamma emitter radionuclide for the first step consisting of imaging, and with a particle emitter radionuclide for the second step (therapy). The imaging procedure, in this case [68Ga]Ga-PSMA PET, helps to define the over-expression of the target, PSMA, in the tumor compared to normal tissues. This total body in vivo evaluation allows an assessment to take place of tumor heterogeneity that is not seen on a single biopsy. If the dosimetry is favorable (based on good biodistribution and prolonged tumor retention), the therapeutic step is possible using particle emitter isotopes (alpha emitters such as Actinium 225 or electrons such as Yttrium 90 or Lutetium 177) to radiolabel the ligands. This allows patients to be selected for PSMA RadioLigand Therapy (PRLT). The same two steps are currently used to select patients for peptide endocrine tumors with the theranostic couple [68Ga]Ga-DOTATOC/[177Lu]Lu-DOTATATE.

Many PSMA-targeting vectors have been created over the past decade for imaging with PET (mostly [68Ga]Ga-PSMA-PET-11, [18F]F-DCFPyL, [18F]F-PSMA-1007). In prostate cancer, PSMA PET improves the detection of metastatic disease compared to conventional imaging for the initial staging of high-risk prostate cancer and biochemical recurrence [24,25,26].

[177Lu]Lu-labeled PSMA radioligand therapy using either PSMA-617 or PSMA-I&T ligand ([177Lu]Lu-PRLT) is an emerging treatment modality, exerting its therapeutic effect by delivering targeted [177Lu]Lu β-particle radiation directly to PSMA-overexpressing cells and the surrounding microenvironment. Binding of the high-affinity ligand to the PSMA protein leads to the internalization and retention of [177Lu]Lu-PSMA in the tumor cell, creating single- and/or double-strand DNA damage by the electrons in the targeted cell as well as in the surrounding tumor cells (crossfire phenomenon). Numerous reviews and clinical studies have positively demonstrated tolerance and efficacy for prostate cancer, paving the way for PRLT to become an important addition to the current therapeutic options in several settings [27,28]. Ongoing trials are evaluating its role in early phases of the disease, while two major studies have evaluated the benefit in heavily pre-treated metastatic-castrate-resistant prostate cancer patients.

The ANZUP Cancer Trials Group reported the results of TheraP, a phase II open-label, randomized, multicenter trial that enrolled metastatic-castrate-resistant prostate cancer (mCRPC) patients, aiming to determine the activity and safety of [177Lu]Lu-PSMA compared to cabazitaxel in men with progressive mCRPC previously treated with docetaxel (NCT03392428) [29]. Of 291 men who were screened, 200 were eligible on PET imaging combining [18F]FDG PET and PSMA-PET criteria. Since the presence of [18F]FDG-positive/PSMA-negative disease was an exclusion criterium for this trial but not used in most other series, the results obtained in TheraP may not be reproduced in another series that do not use both tracers for patient selection. PSA50 was significantly higher with [177Lu]Lu-PSMA-617, 66% versus 37%, while grade 3–4 adverse events were lower, 35% versus 54%. In seven patients who demonstrated an exceptional response based on PSA and complete metabolic response on post-treatment scintigraphy, treatment was paused; however, patients were allowed to restart PRLT up to a maximum of six cycles upon subsequent progression. Progression-free survival at 12 months was 19% (95% CI 12–27) in the [177Lu]Lu-PSMA-617 group compared with 3% (1–9) in the cabazitaxel group. The time to pain progression also favored [177Lu]Lu-PSMA-617 over cabazitaxel, as well as greater improvements in fatigue, social functioning, and insomnia, making [177Lu]Lu-PSMA-617 more suitable for frail patients related to their age or comorbidities.

The international phase III VISION trial (Endocyte, NCT03511664) enrolled 831 mCRPC patients who were randomized in a 2:1 ratio to receive either six cycles of 7.4 GBq of [177Lu]Lu-PSMA-617 plus best supportive/best standard of care (SOC) (n = 551) or SOC-only (n = 280) [30]. The difference in OS was statistically significant, with an estimated 38% reduction in risk of death in the [177Lu]Lu-PSMA-617 arm compared to the best standard-of-care-only arm (median 15.3 months versus 11.3 months, hazard ratio 0.62, *p* < 0.001). Serious drug-related treatment emergent adverse events occurred in 9.3% of patients in the [177Lu]Lu-PSMA-617 arm and 2.4% in the best standard-of-care-only arm. Acute kidney injury was only observed in 3.0% of the [177Lu]Lu-PSMA-617 arm compared to 2.5% on the SOC-only arm.

## 5. Rational for PRLT Applied to ccRCC

So far, there has been no reported case of PRLT applied to ccRCC. Anti-angiogenic drugs have been developed with great success in this tumor type as described before. Therefore, targeting neovessels with PRLT in ccRCC may be of great interest. Direct tumor cell death by electrons emitted by [177Lu]Lu-PSMA as observed in prostate cancer cells may not happen similarly in ccRCC as PSMA is located only in the neovessels and not on tumor cells.

In this setting, electron emitter isotopes would be preferred to alpha-particle emitter as ^177^Lu electrons have an average penetration range of 2 mm or 5 mm for ^90^Y compared to less than 1 mm for alpha particles. The limitation of electron emitters is their dependence on cell cycle to lead to apoptotic death and tumor oxygenation and the production of free radicals, which are usually low in ccRCC lesions. Alpha particles have the advantage of predominately breaking double-strand DNA [31,32], which accounts for the irreversible cell damage. Even though heterogeneous PSMA expression in neovessels leads to the intra-tumor heterogeneity of radioligand binding, tumor cell death may happen through the crossfire effect and the radiation-induced bystander effect (RIBE), which at least partially compensates for the heterogeneity [33,34]. Although the exact mechanism of RIBE is not fully understood, there is evidence that stress signal factors transmit information from irradiated cells to neighboring cells. Moreover, as immune response is highly active in renal cancer, it may be stimulated by radiotherapy, inducing an abscopal effect, which is considered a systemic anti-tumor immune response. Altogether, non-target effects include the effects observed in cells close to the irradiated cells and also long-distance ones [35].

Patients presenting with significant tumor PSMA expression evaluated on the PSMA PET are addressed to [177Lu]Lu-PSMA treatment based on a theranostic procedure. Patients’ selection for PRLT is based on PSMA uptake intensity compared to normal tissues uptake assessed either on a visual analysis as used in the VISION trial [30] or a semi-quantitative analysis calculating the maximal Standard Uptake Value (SUVmax) in a tumor region of interest, like for the TheraP trail [29]. The necessity of this patient selection has been lightened by the poor responses to PRLT observed in case of low tumor PSMA radioligand uptake on PET [36]. Therefore, there is a need to evaluate the pre-test probability to observe a PSMA expression greater than normal tissues on PSMA PET performed in mccRCC patients.

Numerous studies using PSMA PET in ccRCC patients either for initial staging or the diagnosis of recurrence have demonstrated a greater sensitivity compared to conventional imaging [37,38,39]. It also helped to better identify oligo-metastatic disease [40]. A recent review based on 14 studies using different radioligands targeting PSMA for PET for staging or restaging ccRCC patients has confirmed the high frequency of PSMA expression in this type of renal cancer [41]. The detection rate in the per patient analysis ranged from 84 to 100%, and in the per lesion analysis from 80.5 to 100%. They all reported the superiority of PSMA PET over conventional analysis. PSMA intensity of uptake appeared to correlate with the ISUP score, the presence of a sarcomatoïd or rhabdoid component, and the VEGFR-2/PDGFR-β and HIF-2α expression. A change in therapeutic management would concern 13–43.8% of patients. This confirms the interest of therapies targeting PSMA in RCC as previously described by Gorin and Rowe [42].

SUV reflects the radioligand intensity uptake. Overall survival is poorer when the SUV mean of the total tumor volume is lower than 10 in prostate cancer patients [43]. In a retrospective study with 36 ccRCC patients with preoperative [68Ga]Ga-PSMA-11 PET/CT scan and surgical primary tumors specimens [44], PSMA uptake appeared to be related to tumor aggressivity. Indeed, SUVmax could effectively differentiate WHO/ISUP grades, grades 1–2 SUVmax: 13.4 (7.5–18.4) vs. grades 3–4 SUVmax: 23.9 (18.7–29.5) (*p* < 0.001) and different pT stage (T1-T2 vs. T3-T4, *p* = 0.021); tumor necrosis (negative vs. positive, *p* = 0.004); and sarcomatoid or rhabdoid features (negative vs. positive, *p* = 0.035). PSMA staining on tumor vasculature was more intense and diffuse in high-WHO/ISUP-grade tumors and in tumors with adverse pathological features. There were no false negative lesions on PET but 11 on CT in 10 patients with a renal lesion and potential metastatic disease [39]. The average SUVmax in the primary lesion was 18 (range 3.7–36.5), whilst for metastases the average SUVmax was 19.5 (range 1.5–48). A recent study has shown that if the intensity can be different, the presence of PET PSMA-positive metastases in primitive kidney cancers was correlated with expression at the molecular level in immunochemistry, but intensity and distribution could differ [44].

When the SUVmax of ccRCC lesions on PET PSMA are compared to the FDG uptake intensity, in most cases the values are greater with PSMA, as demonstrated in eight patients reported by Siva [45]. FDG uptake could be increased due to hypoxia and the recruitment of Glut receptors.

## 6. Safety of [177Lu]Lu-PSMA

The safety profile of [177Lu]Lu-PSMA-617 and [177Lu]Lu-PSMA-I&T (PSMA-1) is well documented. No life-threatening or disabling adverse events were observed after PRLT, and there were no clinically significant changes in the physical examination of patients with prostate cancer.

As PSMA is physiologically highly expressed in the proximal tubules of normal kidney, the kidneys receive high radiation-absorbed doses. So far, no major renal impairment has been reported after PRLT in prostate cancer patients without the need of amino acid infusion for renal protection, which is the norm with the Somatostatin receptor targeting RLT since its introduction in 1999.

Renal toxicity may be a key issue in patients with one remaining kidney. Interestingly, a recent study has shown the feasibility and tolerability of [177Lu]Lu-PSMA-617 in 16 prostate cancer patients with a single functioning kidney [46]. This study revealed no CTCAE grade 3 or 4 nephrotoxicity and no evidence of any significant hematologic toxicity during or after PRLT in patients with a single functioning kidney. More recent studies confirm the low impact of repeated cycles of PRLT on renal function even in a population with compromised baseline kidney function [47].

The same is observed with [177Lu]Lu-PSMA-I&T (PSMA-1). A study of 100 patients with prostate cancer treated with a cumulative 319 cycles of [177Lu]Lu-PSMA-I&T reported no grade 3 or 4 non-hematologic toxicities. Specifically, no nephrotoxicity was reported in any of the patients with a median follow-up of 9.5 months (range: 7–16.3 months) [48], even though dosimetry studies have demonstrated a greater kidney uptake compared to [177Lu]Lu-PSMA-617 [49].

Indeed, changes in the PSMA ligand can lead to different kinetics and ligand uptake in normal tissues. For example, EB-PSMA-617 was developed to improve the pharmacokinetics of the radionuclide therapeutic agent by extending its blood half-life and tumor residence time. It exhibited significantly higher uptake in xenograft tumors than PSMA-617. The study described an increased kidney uptake that led to increased kidney dosimetry but also increased intra-tumoral residence time compared to [177Lu]Lu-PSMA-617 [50].

In a recent study, the kidney-absorbed dose for [177Lu]Lu-PSMA-617 and [177Lu]Lu-PSMA-I&T differs, with a ~1.5x higher median kidney-absorbed dose for [177Lu]Lu-PSMA-I&T. However, this difference in the clinical setting is considerably smaller than that observed in preclinical studies and may explain the absence of additional renal toxicity when using [177Lu] Lu-PSMA-I&T rather than [177Lu]Lu-PSMA-617 [51].

The safety data are therefore well documented in prostate cancer patients, but the same cannot be said for in renal cancer patients.

## 7. First Trials of Radionuclide Theranostic Applied to ccRCC

So far, there are no data available for PRLT in ccRCC. The first results of safety are pending from a trial conducted in China in which 40 mRCC patients were included and received one injection of 1.85GBq (50 mCi) of [177Lu]Lu-EB-PSMA-617 for(NCT05170555).

The Léon Bérard Cancer Center will be the promoter of an open-label phase I/II study conducted according to a Fleming design, investigating the safety and efficacy of ^177^Lu-PSMA-I&T repeated cycles in patients with metastatic ccRCC. It is divided into two parts. The first is a safety run-in part aiming to assess the safety of [177Lu]Lu-PSMA-I&T (with six patients treated at the starting dose = four cycles of 7.4 GBq of [177Lu]Lu-PSMA-I&T, every 6 weeks). If more than one patient experiences severe toxicity during the first cycle of therapy, then a lower dose of [177Lu]Lu-PSMA-I&T will be evaluated in an additional cohort of six patients. The six patients enrolled in this safety run-in step and treated at the selected dose for phase II will be included in the evaluation of the phase II part. The phase II part will assess the clinical activity of four cycles of [177Lu]Lu-PSMA-I&T. We will also investigate the capacity of [68Ga]Ga-PSMA PET to predict [177Lu]Lu-PSMA-I&T tumor response. Translational studies will consist of comparing efficacy and toxicity to the dosimetry of [177Lu]Lu-PSMA-I&T in normal tissues and tumor lesions, as well as the biomarkers obtained from blood or tumor tissue samples.

**Figure 2:**
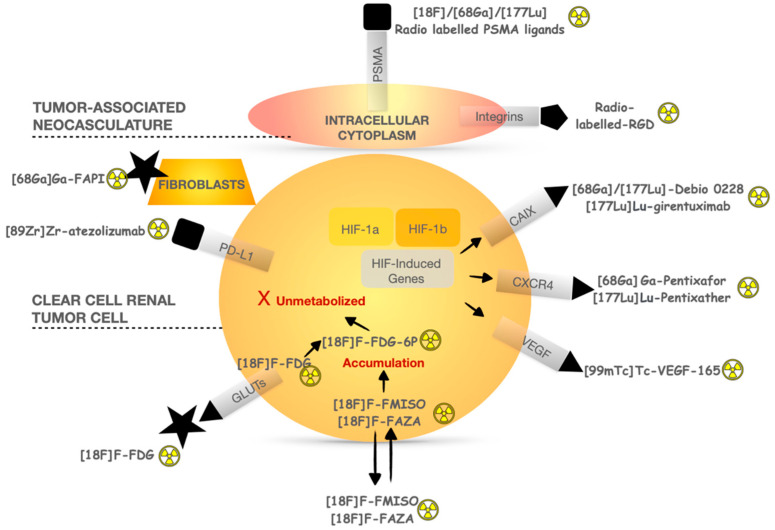
Pathophysiological mechanisms of main radiopharmaceuticals used in ccRCC investigation.

Apart from PSMA, several radiopharmaceuticals have been developed to explore the pathophysiological mechanisms in ccRCC (Figure 2). CAIX is a cell surface glycoprotein expressed in >90% of ccRCC but rarely in normal tissues, providing a target for imaging and therapeutic application. An anti-CAIX monoclonal antibody called girentuximab radiolabeled with Zirconium 89 has shown promising results as a novel PET tracer It can also be labeled with [177Lu]Lu as a therapeutic agent. A phase II study (NCT02002312) included 14 patients treated with a first injection of 2405 MBq/m^2^ of [177Lu]Lu-girentuximab intravenously. In the absence of persistent toxicity and progressive disease, patients could be retreated after 3 months with 75% of the previous activity dose [52]. After the first therapeutic infusion, eight patients (57%) had stable disease and one had a partial response. The treatment was generally well tolerated but resulted in grade 3–4 myelotoxicity in most patients. After the second cycle, continued SD was observed in five of the six patients, but none were eligible for retreatment due to prolonged thrombocytopenia.

An ongoing trial (NCT05239533) is evaluating the safety of [177Lu]Lu-girentuximab associated with nivolumab. Treatment consists of ^177^Lu-girentuximab every 12–14 weeks for a maximum of three doses plus nivolumab 240mg every 2 weeks until disease progression or unacceptable toxicity. Due to expected cumulative myelosuppression, each subsequent [177Lu]Lu-girentuximab dose to the same patient is reduced by 25% (dose 2 = 75% of dose 1; dose 3 = 75% of dose 2).

C-X-C Motif Chemokine Receptor 4 (CXCR4) is part of the human chemokine system and is involved in the progression and metastasis development of renal cell carcinoma (RCC). However, the role of CXCR4 protein expression in RCC remains controversial, and its nuclear or cytoplasmic location appears to be heterogenous between primary and metastases in renal cell cancer as well as in variant renal histologies and benign lesions. CXCR4 expression in ccRCC was not prognostic of overall survival in the multivariate analysis [53]. Radioimmunotherapy targeting CXCR4 with specific radionuclide antibodies appears more interesting in malignancies such as multiple myeloma once again with DLT being hematotoxic [54].

Bone marrow toxicity tends to be the DLT in radioimmunotherapy. The efficacy of antibodies is limited by a longer circulation half-life, which contributes to a high marrow radiation dose and toxicity, as well as poor tumor penetrability, particularly for bone metastases. This was first observed when using radiolabeled anti-PSMA antibodies, mostly J591 for prostate cancer [55], explaining the development and current use of small molecules (PSMA-ligands) for PRLT. Moreover, they can be radio-labeled with a similar high tumor/background ratio and, when compared to antibodies, have more favorable biodistribution with less myelotoxicity.

## 8. Conclusions

Radiolabeled therapy is an emergent anti-cancer treatment as [177Lu]Lu-labeled PSMA ligands have demonstrated promising and encouraging results in metastatic prostate cancer. Radionuclide-therapy-targeting ccRCC neovessels may be of great interest as the use of anti-angiogenic therapy has remarkably improved the prognosis of metastatic RCC patients in recent decades. [177Lu]Lu-PSMA treatments of metastatic prostate cancer appear to be safe. The safety data are well documented, but none of them have been evaluated in renal cancer. Therefore, prospective studies are required. For these reasons, we propose to conduct a phase I/II study evaluating the tolerability and efficacy of [177Lu]Lu-PSMA-1 in patients with PSMA-positive metastatic ccRCC selected through [Ga68]Ga-PSMA PET.

## Data Availability

The data presented in this study are available on request from the corresponding author. The data are not publicly available due to ethical restriction.
